# Risk of heart failure and edema associated with the use of pregabalin: a systematic review

**DOI:** 10.1186/2046-4053-2-25

**Published:** 2013-05-04

**Authors:** Joanne M Ho, Andrea C Tricco, Laure Perrier, Maggie Chen, David N Juurlink, Sharon E Straus

**Affiliations:** 1 Department of Medicine, University of Toronto, Suite RFE 3-805, 200 Elizabeth Street, Toronto, Ontario, M5G 2C4, Canada; 2 Li Ka Shing Knowledge Institute, St. Michael’s Hospital, 30 Bond Street, Toronto, Ontario, M5B 1W8, Canada; 3 The Institute for Clinical Evaluative Sciences, G1 06, 2075 Bayview Avenue, Toronto, Ontario, M4N 3M5, Canada; 4 The Sunnybrook Research Institute, 2075 Bayview Avenue, Toronto, Ontario, M4N 3M5, Canada; 5 Department of Pediatrics, University of Toronto, The Hospital for Sick Children, Room 1436D, 555 University Avenue, Toronto, Ontario, M5G 1X8, Canada; 6 Institute of Health Policy, Management and Evaluation, University of Toronto, Health Sciences Building, 155 College Street, Suite 425, Toronto, Ontario, M5T 3M6, Canada; 7 Division of Geriatric Medicine, University of Toronto, 30 Bond Street, Toronto, Ontario, M5B 1W8, Canada; 8 Office of Continuing Education and Professional Development, Faculty of Medicine, University of Toronto, 500 University Avenue, Suite 650, Toronto, Ontario, M5G 1V7, Canada

**Keywords:** Edema, Heart failure, Observational studies, Pregabalin, Randomized controlled trials, Systematic review

## Abstract

**Background:**

Pregabalin is used in the treatment of postherpetic neuralgia, diabetic neuropathic pain, partial seizures, anxiety disorders and fibromyalgia. Recognized adverse effects associated with its use include cognitive impairment, somnolence and dizziness. Heart failure associated with pregabalin has been described, however the strength of this association has not been well characterized. To examine this further, we will conduct a systematic review of the risk of heart failure and edema associated with use of pregabalin.

**Methods/design:**

We will include all studies (experimental, quasi-experimental, observational, case series/reports, drug regulatory reports) that examine the use of pregabalin compared to placebo, gabapentin or conventional care. Our primary outcome is heart failure and the secondary outcomes include edema and weight gain. We will search electronic databases (MEDLINE, EMBASE, Cochrane Central Register of Controlled Trials), and grey literature sources (trial registries, conference abstracts) to identify relevant studies. To ensure literature saturation, we will contact drug manufacturers, conduct forward citation searching, and scan the reference lists of key articles and included studies. We will not restrict inclusion by language or publication status.

Two reviewers will screen citations (titles and abstracts) and full-text articles, conduct data abstraction, and appraise risk of bias. Random-effects meta-analysis will be conducted if the studies are deemed heterogeneous in terms of clinical, statistical and methodological factors but still suitable for meta-analysis.

**Conclusions:**

The results of this review will assist physicians to better appreciate pregabalin’s risk for edema or congestive heart failure and will be pertinent to the thousands of patients worldwide who are administered this medication.

Our protocol was registered in the PROSPERO database (CRD42012002948).

## Background

Pregabalin, a structural analogue to gamma-aminobutyric acid (GABA), is a new medication that is widely prescribed for chronic pain syndromes such as postherpetic neuralgia and diabetic neuropathic pain, as well as partial seizures, anxiety disorders and fibromyalgia [1]. It is a calcium channel antagonist that decreases the release of several neurotransmitters including substance P, norepinephrine and glutamate, without binding to GABA receptors; however, its mechanism of action is still not well understood [1].

Pregabalin’s known adverse effects include cognitive impairment, somnolence and dizziness [2]. Post-marketing surveillance has also noted an increasing number of reports of heart failure in patients using the drug, an adverse outcome that has not been found with the less potent calcium channel antagonist gabapentin [3-6]. A systematic review of randomized controlled trials involving pregabalin found a 4-fold increased incidence of peripheral edema, which may be associated with heart failure. Since these trials included patients who were healthier and more closely monitored than the general population, this risk of edema or heart failure may actually be higher [7]. Furthermore, individuals for whom pregabalin is often prescribed, such as diabetic patients, tend to have renal or cardiac disease, which are known risk factors for heart failure [8]. In clinical practice, the high background incidence of edema and heart failure may reduce the likelihood that health care providers attribute these problems to a medication.

The risks associated with pregabalin may be better studied by examining observational studies, which include greater numbers of patients with comorbid conditions – studies not included in previous reviews [7]. We hypothesize that there is an increased risk of heart failure or edema in individuals receiving pregabalin compared to placebo or gabapentin. To investigate this further, we will conduct a systematic review of pregabalin across all available studies. Our research question is “what is the risk of heart failure or edema among patients newly started on pregabalin compared to gabapentin, placebo or conventional care?”

## Methods

This research protocol was developed by our team with expertise in geriatric medicine, clinical pharmacology, systematic review methodology, statistics and library science. Our protocol was registered in the PROSPERO database (CRD42012002948).

### Eligibility criteria

We will include any study report that examines congestive heart failure, edema or weight gain among adult patients (age ≥18 years) newly prescribed pregabalin compared to gabapentin, placebo or standard medical care. Studies will be included regardless of publication status or language of dissemination. We will exclude qualitative studies but all other study designs will be included. The PICOST criteria are as follows:Patients – : Adults ≥18 years; Intervention – : Pregabalin (any dosage and duration of use); Comparator – : Gabapentin (another calcium channel antagonist) placebo or conventional medical care; Outcome – : Heart failure (primary) edema weight gain alone or with edema (secondary); Studies – : Clinical trials (randomized clinical trials, quasi-randomized clinical trials, controlled clinical trials), quasi-experimental studies (controlled before-after studies and interrupted time series), observational studies (case–control, cohort, case-crossover, case-time-control), case series/reports, safety bulletins and primary surveillance data from drug regulatory agencies; Time – : No restrictions will be imposed based upon study duration; Other – : No other restrictions will be imposed (*e.g.*, language publication status or year of dissemination)

### Information sources

An experienced librarian (LP) will conduct the literature search and the search strategy will be peer reviewed by another librarian using the Peer Review of Electronic Search Strategies (PRESS) checklist [9]. We will search the following electronic databases from inception onwards: Medline (1946 to present), EMBASE (1947 to present), Cochrane Central Register of Controlled Trials (CENTRAL). The full search strategy for the main electronic search in MEDLINE is presented in Appendix A. We will use the World Health Organization International Clinical Trials Registry Platform search portal of trial protocols to simultaneously search multiple trial registry sites (http://www.controlled-trials.com/mrct/) and conference abstracts (*e.g.*, American Academy of Pain Medicine annual meeting, American Pain Society annual meeting, International Congress for Neuropathic Pain, American Academy of Neurology annual meeting, American Diabetes Association annual meeting, Canadian Diabetes Association annual meeting) for difficult to locate or unpublished (*i.e.*, grey) literature. The following Regulatory Authority safety alerts will be searched: Food and Drug Administration MedWatch (United States), European Medicines Evaluation Agency's European Public Assessment Reports, Medicines and Healthcare Products Regulatory Agency (United Kingdom), Australia Adverse Drug Reactions Bulletin, Health Canada MedEffect and the Canadian Adverse Drug Information System. We will also search the World Health Organization Uppsala Monitoring Centre.

### Study selection

The eligibility criteria will be pilot-tested on a random sample of 50 citations, which will be screened by the entire team. A kappa statistic will be calculated to measure inter-rater reliability and screening will only commence when ≥60% agreement is achieved [10]. Using the online SysRev Tool (proprietary software available at the Li Ka Shing Knowledge Institute of St. Michael’s Hospital), two reviewers will subsequently screen the literature search results at citation (titles and abstracts) and full-text article levels in duplicate. Conflicts will be resolved by discussion between the reviewers or with a third reviewer, if necessary.

Some of the included study reports might be studies examining pregabalin among the same patient population (*i.e.*, companion reports). To identify studies that generate multiple reports (duplication bias), we will record the authors' names, study location and setting, dose and frequency of pregabalin administration (intervention), number of participants and their baseline demographic data, and date and duration of the study. Once identified, we will link these reports. We will consider the report with the longest duration of follow-up or primary outcome of interest as the major publication and the rest will be considered companion reports which provide supplementary information.

This is a systematic review of adverse events, which are often underreported in randomized clinical trials [11]. To ensure that trials not reporting this information are not systematically different compared to those that do (*i.e.*, outcome reporting bias [12]), we will contact authors of trials that do not report our outcomes of interest. We will exclude non-randomized studies that do not provide data on our primary or secondary outcome.

### Data collection process

A data abstraction form will be developed and amended following a pilot-test on a 5% random sample of the included studies by all reviewers. Two reviewers will subsequently perform all data abstraction in duplicate. Conflicts will be resolved by discussion or, if necessary, a third reviewer will be involved. We will attempt to contact study authors to verify data, as necessary. The anticipated data that will be collected are included in Appendix B.

### Risk of bias

For randomized clinical trials, we will use the Cochrane Collaboration's 5.1.0 risk of bias tool. This 7-item tool assesses for selection bias, performance bias, detection bias, attrition bias, and reporting bias [13].

For non-randomized studies and observational studies, a single risk of bias tool has not been validated [14]. As such, we will use a combination of the Newcastle-Ottawa Scale, the Effective Practice and Organization of Care Risk of Bias Tool and the McMaster Quality Assessment Scale for Harms [15-17]. We will apply the Naranjo Probability Scale for case reports/series [15-19].

### Confounding variables

The following are variables that are confounders for pregabalin and the risk for edema or heart failure:

1. Demographics: Age, gender, sociodemographic status/income

2. Comorbidities: Diabetes, hypertension, dyslipidemia, congestive heart failure, cardiovascular disease, cerebrovascular disease, renal disease, seizure disorder, number of medications per year, burden of illness scales (Charlson, Romano, Aggregated Diagnosis Groups, recent hospitalization in the past 12 months)

3. Medications that affect fluid balance: Loop diuretics

4. Cardiovascular medications: Renin-angiotensin antagonists (angiotensin converting enzyme inhibitors, angiotensin receptor blockers, aliskiren), negative chronotropes (beta blockers, calcium channel blockers, digoxin), statins, antiarrhythmics, antiplatelet agents, anticoagulants

5. Medications that can exacerbate or trigger congestive heart failure: Non-steroidal anti-inflammatory drugs, steroids, thiazolidinediones

6. Medications associated with severe neuropathic pain: Opioids

7. Medications associated with other indications for pregabalin: Anticonvulsants, benzodiazepines, antidepressants

We will record which studies adjusted for which variables, and their crude and adjusted measures of effect size (*e.g.,* odds ratio, relative risk).

### Synthesis of results

Study heterogeneity will be examined using Q- and I^2^-statistics [20]. If the studies are clinically, statistically and methodologically homogenous, a meta-analysis will be conducted separately for randomized clinical trials and cohort studies using a random-effects model [21]. The relative risk will be calculated for the occurrence of heart failure, edema and weight gain (alone or with edema) from randomized clinical trials, while odds ratios will be calculated for these outcomes from cohort studies. Furthermore, a network meta-analysis may be considered with randomized clinical trials that compare pregabalin or gabapentin with placebo. If at least 10 studies are included in the meta-analysis, publication bias will be assessed using a funnel plot [22]. Extensive heterogeneity, defined as I^2^ >60%, will be addressed with sub-group analysis or, if there are >10 studies reporting relevant outcomes, meta-regression will be considered. Variables that will be examined further using sub-group analysis and/or meta-regression include pregabalin dose, patient age and history of postherpetic neuralgia and of diabetic neuropathy. The methodological quality and risk of bias results will be scrutinized and sub-group analysis will be conducted on those items that are of low methodological quality for the cohort studies or high risk of bias for the randomized clinical trials. The results of case series, case reports, and case–control studies will be summarized descriptively and will not be meta-analyzed.

## Discussion

Through this systematic review, we will gain a better appreciation of pregabalin’s risk of heart failure and edema. The results of this review will be of interest to clinicians and the thousands of patients worldwide who are administered this heavily marketed medication. Given that pregabalin is not universally covered by health care plans in North America, this systematic review may also be helpful to health policy makers should they consider including this medication in drug benefit programs.

Our knowledge exchange strategies include a publication in a peer-reviewed journal and presentations at upcoming meetings, such as the Drug Safety and Effectiveness Network meeting in Canada.

Finally, this is the first phase of a more comprehensive network meta-analysis of the comparative harms of non-opioid analgesics, a clinically relevant topic to clinicians, patients and health policy makers.

## Appendix A

### Search strategy

Medline (1946 to present):

1. gamma-Aminobutyric Acid/aa [Analogs & Derivatives]

2. pregabalin.tw.

3. lyrica.tw.

4. "3-isobutyl GABA".tw.

5. pregablin.mp.

6. CI-1008.tw.

7. S1731_Selleck.tw.

8. "3-(aminomethyl)-5-methylhexanoic acid".tw.

9. 148553-50-8.rn. [CAS Registry Number]

10. "3 isobutyl 4 aminobutyricacid".tw.

11. "3 isobutylgaba".tw.

12. "4 amino 3 isobutylbutyric acid".tw.

13. "pd 144723".tw.

14. or/1-13

15. exp Adult/ [ adult filter - validated, highly sensitive ]

16. adult.mp.

17. Middle Aged/

18. age$.tw.

19. or/15-18

20. 14 and 19

21. exp Animals/ not (exp Animals/ and Humans/) [ removing animal studies ]

22. 20 not 21

## Appendix B

### Data collection

The following data categories will be collected:

1. Patient characteristics:

1. Total number (baseline, study end)

1. Setting

1. Diagnostic criteria

1. Age (median, interquartile range)

1. Gender (% female)

1. Country

1. Co-morbidities

1. Socio-demographics

1. Ethnicity

1. Date of study

2. Study characteristics:

1. Report ID (created by review author)

1. Citation and contact details

1. Confirm eligibility for review

1. Reason for exclusion

1. Study design

1. Country

1. Setting (outpatient, inpatient)

1. Publication status

1. Intervention and comparator descriptions (dosage, intensity, frequency)

1. Allocation to groups (concealed randomization, quasi-randomization, time differences, location differences, policy/public health decisions, cluster/individual preferences)

1. Prospective (Whole *vs.* components)

1. Duration of intervention and follow-up

1. Outcomes examined

1. Variables that were assessed between groups (potential confounders, baseline assessment of outcome variables)

1. Funding source

1. Key conclusions of the study authors

1. Miscellaneous comments from study authors

3. Quality/Risk of bias:

1. Cochrane 5.1 collaboration's tool for assessing risk of bias categories (randomized clinical trials)

1. Newcastle-Ottawa Scale for assessing risk of bias categories (cohort and case–control studies)

1. Effective Practice and Organization of Care (EPOC) for assessing risk of bias (controlled clinical trials, controlled before-after trials and interrupted time series)

1. Naranjo Adverse Drug Reporting Probability Scale (case reports/series)

1. McMaster Quality Assessment Scale for Harms Study (McHarm)

4. Adverse events (systematic review primary outcome – congestive heart failure; secondary outcomes – edema, weight gain)

1. Diagnostic criteria

1. Number of events in intervention and control/comparison groups

1. Sample size

1. Frequency

1. Relative risk (randomized trials)

1. Odds ratio (cohort studies)

1. Severity

1. Resulted in withdrawals

1. Collected at follow-up (frequency of follow-up)

1. Collected by patient diary or checklist or spontaneous reporting

1. Early *vs.* late withdrawal

1. Other adverse events reported

## Competing interests

The authors declare that they have no conflicts of interest.

## Authors’ contributions

JMH conceived the study, designed the study, helped develop the search strategies, registered the protocol and helped write the draft protocol. ACT helped design the review methods, and edited the draft protocol. LP helped develop the search strategies and edited the draft protocol. MHC provided input to the review conceptualization and edited the draft protocol. DNJ helped conceive the study and edited the draft protocol. SES helped conceive and design the study, and edited the draft protocol. All authors read and approved the final protocol.

## Authors’ information

JMH is a practicing internist, geriatrician and clinical pharmacologist, and trainee with the University of Toronto Eliot Phillipson Clinician Scientist Training Program and Li Ka Shing Knowledge Institute. ACT is a systematic review methodologist and co-directs a knowledge synthesis center with SES. DNJ is a practicing internist and clinical pharmacologist, and Division Head of Clinical Pharmacology at the University of Toronto. SES is a practicing internist and geriatrician, and Division Head of Geriatric Medicine at the University of Toronto. MC is a biostatistician with the Li Ka Shing Knowledge Institute. LP is an information specialist with the Li Ka Shing Knowledge Institute and the University of Toronto.
